# Oral health of the 65+ age group in Israel-2020

**DOI:** 10.1186/s13584-021-00494-6

**Published:** 2021-10-25

**Authors:** Ayelet Berg-Warman, Ile Kermel Schiffman, Shlomo P. Zusman, Lena Natapov

**Affiliations:** 1 Myers-JDC-Brookdale Institute, Jerusalem, Israel; 2 Division of Dental Health, Ministry of Health, Jerusalem, Israel

**Keywords:** Oral health, Natural teeth, Edentulous, 65+ years of age

## Abstract

**Background:**

In 2019, a reform of dental services for older adults was implemented in Israel to improve access and reduce barriers that stood in their way. The reform stipulated that preventive and restorative dentistry would be included in the basket of services of the National Health Insurance Law. The current study was conducted by the Myers-JDC-Brookdale Institute (MJB) and the Division of Dental Health of Israel’s Ministry of Health to examine the dental status and patterns of utilizations of dental services among the 65+ age group. This paper reports on the dental status of the 65+ age group in comparison with the same population two decades earlier.

**Goals:**

To describe the dental status of Israel’s 65+ age group, and to identify the population at risk of dental morbidity.

**Methodology:**

Telephone interviews were conducted with a representative sample of 512 older adults aged 65+, from February to April 2020.

**Main findings:**

Some two-thirds of the 65+ age group assessed their oral health as good or very good. Twenty-four percent did not have natural teeth, while the rest had 19 teeth on average. Ten percent had not lost any teeth. In the 65–74 age group, 19% had no natural teeth and the rest had 20 teeth on average. In contrast, in the 85+ age group, 38% were edentulous and the rest had 13 teeth on average. Of the older adults who found it difficult to cover their monthly expenses, 39% were edentulous—twice the percentage of those who did manage to cover their monthly expenses (19%). Of the 65+ age group 44% had dentures—37% in the 65–74 age group, and 66% in the 85+ age group. Approximately 40% of the 65+ age group saw a dentist for preventive check-ups. The rest did not, mainly due to lack of awareness of the importance of doing so.

**Conclusions and recommendations:**

The perceived status of oral health among the 65+ age group is currently better than it was 22 years ago. However, despite the improvement in oral health and health behavior, there are still barriers to the utilization of dental services. The main barriers are a lack of awareness of the importance of proper health behavior, and the cost of care for people with financial difficulty. This study provides decision-makers with data on the status of oral health, the utilization of dental services and the geographical disparities. The findings will help policy makers evaluate the effectiveness of the reform and fine tuning it in the future. Policies should be instated to increase awareness of constituencies and their access to the services, in addition to the entitlements the reform granted.

## Introduction

Oral health considerably impacts the quality of life and nutrition of older adults. Healthy teeth contribute to the ability to chew, swallow, and speak. The physiological changes that come with aging may include poor health of teeth and gums or chewing problems that affect the consumption of proteins, nutritional fibers, and vegetables, to the detriment of the elders’ physical condition. Oral health also affects a person's appearance and as a result—the extent of social involvement of the older population, as well as their self-image.

International comparative studies have shown that the oral health of Israel’s older adults is inferior to that of their peers in many developed countries [6, 9, 16]. Partially, the explanation lies in the barriers that hamper Israel’s older adults from taking up dental services. Studies conducted in Israel have emphasized financial barriers and the importance of adding preventive, conservative, and restorative dentistry to the basket of services under the National Health Insurance Law, and with reference to lack of awareness, preventive behavior was left addressed.

In 2019, dental services for Israel’s older adults were reformed to improve access and reduce some of the barriers that stood in their way of their uptake. The reform stipulated that as of February 2019, preventive and conservative dentistry would be added to the universal basket of services for the 75+ age group, and from October 2019, restorative dentistry (prosthodontics) would be added for the 80+ age group.

Oral health is vital to good quality of life [15] as dental and periodontal problems affect the ability to eat, social life, and quality of sleep. Access to dental services is important to preserve oral health,those at risk of high dental morbidity are usually low-income groups or members of minority groups, as well as immigrants, the housebound, and institutionalized older adults [2, 11, 20].

Studies that have examined the oral health of Israel’s older adults showed that about 50% assessed their oral health as good or very good [6, 12]. A national study conducted in 1998 [6] found that 52% of the 65+ age group had lost all their teeth. The study pointed to gaps in morbidity, by financial status—some 70% of the 65+ older adults in the bottom third income percentile had lost all their teeth versus 45% in the top third income percentile. The study also showed that some 88% of the low-income 65+ age group reported that they had false teeth on at least one jawbone versus 63% of the high-income older adults [5].

The first national survey of the state of health and nutrition (SHN)^1^ among the 65+ age group, conducted by the Ministry of Health in 2005–06, showed that the general health of 17% of Israel’s older adults was affected by their oral health status [22]. Considerable differences were found in the consumption of energy, proteins, nutritional fibers, and vegetables according to the oral and dental health status of the examinees. Thus, among those who reported chewing problems due to their teeth, a lower consumption of nutritional components was found. Moreover, the consumption of various nutritional elements by older adults with artificial teeth was lower than that of older adults with natural teeth (21 natural teeth and 4.1 pairs of teeth on average) [17]. To date, there has been no clinical assessment of the state of oral health of the 65+ age group in Israel.

Mann et al. [14] showed that among the mixed population of institutional residents and older adults in day care centers, some 63% had no teeth on one jaw, and 60% had no teeth on both jaws. Adut et al. [1] found that 54.4% of the 65+ age group living in the community had no teeth, and the rest had 10.4 teeth on average. The highest percentage of people with no teeth at this age was found among the Arab population (67.2%).

There is little comparative, international information on oral health that is both up to date and reliable. An earlier international comparison indicated that the condition of oral health of Israel’s older adults was inferior to that of their peers in many developed countries according to the percentage of people with tooth loss and the average number of teeth. One of the few comparative studies published showed that in the 1990s, the percentage of people aged 75+ and missing teeth was: 27% in Sweden, 45% in Denmark, and 58% in Finland [16]. Another comparative study from Australia showed that in 2004–06, in the 65–74 age group, 20.3% had no teeth, and in the 75+ age group, 35.7% had none. In Germany, in the 65–74 age group, 22.6% had no teeth [9]. In Britain, the percentage of edentulous people in the 65+ age group dropped from 28% in 1978 to 6% in 2009 [19]. Based on data from 2011 to 2016, the percentage of edentulous people in the US among the 65+ age group was 17.3% [11], among the 65–74 age group, it was 13% and among the 75+ age group, the percentage was—26% [20].

Another measure of oral health is the number of teeth. In the US, the average number of teeth in the 65–74 age group is 23.7, and in the 75 + age group—22.8; in Australia these figures are 22.9 and 21.0, respectively; in Germany, in the 75 + age group—17.3 [9]. In the US State of Ohio, 50.6% of the 65+ age group have fewer than 20 teeth, and 28.6% of this age group have no teeth [13]. The World Health Organization has set the number of 20 natural teeth needed for normal functioning, enabling comfortable eating and socializing without embarrassment—a target that many countries have yet to reach [16]. Little is known about the state of dental health of older adults in other countries in recent years.

This study examines the state of dental health and patterns of use of dental services in Israel among the 65+ age group. It focuses on this group in comparison to their staus two decades ago, and to that of peers in other countries. We hope that the results will help policymakers better adapt the provision of dental services to the population of older adults in this era of reform, and shed light on the extent of existing gaps between different population group based on financial status and geographic location.

## Study goals

- To learn about the oral health status, attitudes and oral health behavior of Israel’s older adults in 2020
- To identify the population at risk of dental morbidity and the main barriers to their uptake of dental services in 2020
- To provide additional dataset to the national oral health data.

## Methodology and materials

The study population consisted of the 65+ age group in Israel living in the community—about a million people.

### The sample

A random sample of 1250 people aged 65+ was drawn from computerized telephone books. The interviews were conducted between February and April 2020. In cases where the older adults themselves could not be interviewed for reasons of health or cognition, relatives were interviewed about them. They were asked objective questions about the older adults, such as the number of teeth they had and whether they visited a dental clinic for check-ups.

### The research tool

The questionnaire was constructed from the International Collaborative Study I and II of the World Health Organization—WHO [8] and the World Dental Organization—FDI, in the US and Europe [3], National Institute of Dental Research [18]. It dealt with the following topics: The self-assessment of the older adults of the state of their oral health, their knowledge of healthy oral behavior, and their attitudes to the importance of healthy teeth. The questionnaire was translated into Arabic and Russian to include the main non-Hebrew speaking populations in Israel. It was initially tested on 15 interviewees and subsequently fine-tuned accordingly.

### Data collection

The distribution and comparison of the characteristics examined were calculated by age group and economic status, the latter measured by the self-reported extent of ability to cover monthly household expenses: Whether managing to do so "without difficulty", "managing to do so", or finding it "difficult to do so" (which included not managing at all). Some topics were also compared by gender, residential region (the center of the country versus the periphery^2^), and population group. These comparisons were performed using the χ^2^ test. To compare the average number of natural teeth of the different groups, the t test was used. The association between the self-assessment of health status and the assessment of oral health—was examined using the Spearman coefficient. In addition, a linear regression model was used to explain the condition of oral health with the explanatory variables: background characteristics and health-behavior knowledge and attitudes. *p* < 0.05 was regarded as statistically significant.

### Ethics

The study was approved by the Helsinki Committee of the Ministry of Health (No. 16/2019; 11.7.2019) and the Ethics Committee of the Myers-JDC-Brookdale Institute (23.7.2019). The interviewers asked for the consent of the participants to be interviewed after they had received an explanation on the background to, and goals of, the study. The interviews were conducted after consent had been given.

## Results

Of a random sample of 1250 subjects aged 65+, 64 had died. In 174 cases, the telephone numbers were wrong, and 258 subjects could not be reached by telephone after a maximum of six attempts. Of the 754 subjects aged 65+ that comprised the study population, 512 were interviewed by telephone (67.9% response rate), 168 refused to be interviewed, and 74 were not interviewed for other reasons (such as language barriers, communication problems or the end of the interview phase in the study). The interviews were conducted from February to April 2020. In cases where an older adult could not be interviewed for reasons of health or cognition, a relative was interviewed about them. In total, 34 relatives were interviewed on objective questions about the older adult, such as the number of teeth they had and whether they visited a dental clinic.

### Demographic characteristics

The interviewees were divided into three age groups: 65–74, 75–84, and 85+. Table 1 presents the interviewees’ characteristics, by age group. It shows that 52.0% were women, 71.2% were married, and the average age was 75.6. The 85+ age group contained a higher proportion of women, widows and subjects with a lower education (elementary school or less) than the 65–74 age group. Understandably, the percentage of employed participants in the 85+ age group was lower (3.5%) than that of the 65–74 age group (33.2%).

**Table 1:** Background characteristics, by age (%)

	65–74	75–84	85 +	Total	65+ in the population^a^
n (in sample)	293	148	71	**512**	
N (in population)	626,523	301,850	127,943	**1,056,315**	**1,056,315**
Women	48.5	55.8	58.7	**52.0**	**55.5**
Average age (years)	70.8	79.2	87.5	**75.6**	
(*SD*)	*(2.1)*	*(2.9)*	*(1.6)*	***(6.4)***	
Marital status**					
Married	76.7	68.9	49.2	**71.2**	**71.2**
Widowed	14.4	24.4	44.1	**20.8**	**20.8**
Divorced/single/separated	8.9	6.7	6.8	**8.0**	**8.0**
Education*					
Elementary/junior high school	18.6	35.4	34.4	**25.2**	**17.9**
Vocational high school	12.4	12.0	9.8	**12.0**	**10.8**
High school	14.8	14.3	11.5	**14.3**	**18.9**
Yeshiva	1.7	0.0	0.0	**1.0**	**1.2**
Post-high school	17.6	9.8	14.8	**15.1**	**15.5**
University	34.8	28.6	29.5	**32.4**	**31.6**
Other, not known					**3.9**
*Country of birth*					
Israel	40.9	39.3	28.8	**39.0**	**30.2**
Europe & America	35.9	23.6	39.0	**32.9**	**41.4**
Asia & Africa	23.2	37.1	32.2	**28.1**	**28.4**
Of these: made aliya after 1989	25.2	19.2	26.7	**23.7**	**21.5**
*Religion*					
Jewish	87.5	90.3	93.7	**89.1**	**86.9**
Muslim	7.9	6.2	1.6	**6.6**	**6.3**
Christian	2.0	2.1	4.8	**2.4**	**1.9**
Druze	1.3	0.7	0.0	**1.0**	**0.9**
Other	1.3	0.7	0.0	**1.0**	**4.0**
Employed***	33.2	14.8	3.5	**24.5**	**22.4**
*Ability to cover monthly expenses*					
Without difficulty	10.3	11.4	3.4	**9.8**	**21.4**
Managing	71.8	62.1	74.6	**69.5**	**53.6**
Finding it difficult	17.9	26.5	22.1	**20.7**	**25.0**

The bold numbers are for the Total columns, the italics are for standard deviation

*p < 0.05

**p < 0.01

***p < 0.001

^a^Shnoor and Cohen, The 65+ population in Israel: statistical abstract 2020

### Health condition and characteristics of oral health

The interviewees responses concerning the condition of their teeth and gums are presented in Table 2: 72.9% assessed their general health as good or very good, 21.6%—as not so good, and 5.5%—as not good. When asked to assess the condition of their oral health, 65.5% assessed it as good or very good, 25.0%—as not so good, and 9.5%—as not good. A positive correlation was found between the condition of general health and the condition of oral health: 75% of the subjects who assessed their oral health as good or very good also assessed their general health thus (Spearman = 0.383); 66.7% of the subjects noted that they were satisfied with the condition of their oral health, 19.3%—were not so satisfied, and 14.0% were not satisfied. The main reasons given were pain or difficulty in eating (12% for each), appearance and ongoing problems (5% for each). Few interviewees (3%) were compelled to give up recreational activity in the past year due to problems with their teeth (this does not appear in the table).

**Table 2:** Condition and characteristics of oral health (%) (n = 512)

	Total	74–65	84–75	85 +
Assessment of general health*				
Very good	**22.5**	28.0	14.0	7.9
Good	**50.4**	48.1	54.4	55.3
Not so good	**21.6**	18.8	25.0	31.6
Not good	**5.5**	5.1	6.6	5.3
*Assessment of oral health*				
Very good	**15.4**	17.8	12.2	11.1
Good	**50.1**	46.5	56.5	52.4
Not so good	**25.0**	24.5	24.5	27.0
Not good	**9.5**	11.2	6.8	9.5
*Satisfaction with condition of oral health*				
Satisfied	**66.7**	64.7	69.5	71.1
Not so satisfied	**19.3**	19.2	18.8	22.2
Not satisfied	**14.0**	16.1	11.7	6.7
Number of natural teeth*				
None	**23.9**	18.5	28.8	38.1
All	**9.9**	11.2	13.3	5.6
Average no. of teeth (among those who have teeth)***	**19.1**	20.3	18.4	12.8
(*SD*)	***(10.5)***	*(10.1)*	*(10.8)*	*(10.4)*
Average no. of teeth (among the 65+ age group)***	**14.0**	16.1	12.5	7.3
(*SD*)	***(12.3)***	*(12.2)*	*(12.3)*	*(10.1)*

The bold numbers are for the Total columns, the italics are for standard deviation

*p < 0.05

***p < 0.001

Table 2 shows that 23.9% of the subjects had lost all their teeth, and 9.9% possessed all their teeth. Among the 65–74 age group, 18.5% had lost all their teeth versus 38.1% among the 85+ age group. Of those who had teeth, the average number of natural teeth was 19.1 (i.e., an average of 13 lost teeth per individual). The 65–74 age group had 20.3 teeth on average (a loss of 12 teeth on average per individual); the 75–84 age group had an average of 18.4 teeth (a loss of 14 teeth on average per individual); and the 85+ age group—12.8 teeth (a loss of 19 teeth on average per individual). Some 37.0% of the interviewees had at least 20 teeth, the acceptable level as defined by the WHO for normal functioning (this does not appear in the table).

Table 3 presents the characteristics of the subjects’ oral health, by economic status (i.e., the economic status as measured by the ability to cover monthly expenses). As in other studies, a positive association was found between general health/oral health and economic status. This was corroborated by the individual’s number of teeth—17.4% of those who managed to cover their monthly expenses without difficulty had lost all their teeth while the average number of teeth among subjects who had any was 22. In contrast, 38.6% of those who found it difficult to cover their monthly expenses had lost all their teeth, and the rest had an average of 18 teeth. It thus follows that teeth and gum problems are more prevalent among the latter (data not shown).

**Table 3:** General health and oral health, by ability to cover monthly expenses (%) (n = 482)

	Total^a^	Manage without difficulty	Manage	Find it difficult
Assessment of general health**				
Very good	**22.5**	42.2	23.2	12.9
Good	**50.4**	46.7	50.8	49.4
Not so good	**21.6**	8.9	20.3	31.8
Not good	**5.5**	2.2	5.7	5.9
Assessment of oral health*				
Very good	**15.4**	17.4	16.6	8.9
Good	**50.1**	56.5	50.6	43.6
Not so good	**25.0**	15.2	25.9	29.7
Not good	**9.5**	10.9	6.9	17.8
*Number of natural teeth*				
None***	**23.9**	17.4	18.5	38.6
All	**9.9**	8.9	12.4	4.4
Average no. of teeth (among those who have teeth)	**19.1**	22.2	18.7	18.2
(*SD*)	***(10.5)***	*(9.6)*	*(10.8)*	*(10.4)*
Average no. of teeth (among the 65+ age group)	**14.0**	18.2	14.8	10.3
(*SD*)	***(12.3)***	*(12.2)*	*(12.3*)	*(12.0*)

The bold numbers are for the Total columns, the italics are for standard deviation

*p < 0.05

**p < 0.01

***p < 0.001

^a^Including 30 interviewees who did not answer the question on the ability to cover monthly expenses

No differences were found between men and women; 22.6% of the women and 25.1% of the men had no teeth (*p* = 0.801). Based on their self-reports, the number of teeth among subjects who had any was also similar: women—19.7; men—18.4 (*p* = 0.279). Among those living in peripheral areas, 28.8% had lost all their teeth versus 23.7% of residents in the center of the country (*p* = 0.098). The number of teeth of older adults in the center and in the periphery was similar (*p* = 0.257). In contrast, considerable differences were found between Jews and non-Jews: 20.6% of the Jews had no teeth versus 51.9% of the non-Jews (*p* < 0.001).

Table 4 presents the frequency of teeth and gum problems potentially detrimental to various areas, such as social life and chewing ability: 20.0% of the interviewees noted that their gums were painful or sensitive, 14.3% had bleeding gums, and 11.7% had loose teeth. Because of these and additional problems, apparently, 19.6% of the interviews reported difficulty in chewing solid foods, and 63.5% ate soft food such as cooked cereals and ground foods. A higher percentage of chewing problems was found among the 85+ age group (27.4%, of whom 94.4% ate soft foods).

**Table 4:** Gum and teeth problems, by age (%) (n = 512)

	Total	74–65	84–75	85 +
Painful or sensitive gums	**20.0**	19.7	17.0	29.0
Bleeding gums	**14.3**	17.2	11.6	9.7
Loose teeth	**11.7**	10.6	11.5	18.9
Swollen gums	**10.4**	10.6	8.2	14.5
Chewing problems	**19.6**	17.2	21.2	27.4
Of these: Eat soft foods due to chewing problems*	**63.5**	55.3	58.1	94.4
Bad breath (often or sometimes)	**17.2**	17.8	16.2	15.9

The bold numbers are for the Total columns

*p < 0.05

The data analysis showed that some problems were more common among women and older adults who had difficulty covering their monthly expenses—23.9% of the women reported painful or sensitive gums versus 15.0% of the men (*p* = 0.016). Similarly, 13.3% of the women reported swollen gums versus 7.3%% of the men (*p* = 0.019). Among those who find it difficult to cover their monthly expenses, 28.1% had painful or sensitive gums, and 26.2% had loose teeth versus 17.4% and 8.1% respectively, among those who managed to cover their monthly expenses (*p* = 0.01). Ajjs regards the other problems, no differences were found by financial status (this does not appear in the table.).

Table 5 presents the use of dentures, by age and problems of usage: 43.8% of the interviewees had dentures, a percentage that rises with age; 37.3% of the 65–74 age group reported that they had dentures versus 66.1% of the 85+. Of those with dentures, 75.0% had a full upper denture, 17.4% had partial dentures, 59.4% had a full lower dentures, and 21.9% had partial lower dentures. Of those who had dentures, 90.7% used them all the time or during waking hours, 3.5% used them only for eating, and 4.8% did not use them at all. Subsequently, the subjects noted problems that their dentures caused them: some 10% said that they caused pain or sores, and 20% said that they bothered them when eating (this does not appear in the table).

**Table 5:** Use of dentures by age (%) (n = 512)

	Total	74–65	84–75	85 +
Has dentures***	**43.8**	37.3	47.9	66.1
*Of these*:				
Full uppers	**75.0**	71.8	77.5	79.1
Partial uppers	**17.4**	20.0	12.7	18.6
Full lowers	**59.4**	54.1	63.4	65.9
Partial lowers	**21.9**	22.9	22.5	18.2
*Frequency of use of dentures*				
All the time	**48.0**	50.4	47.9	41.9
Waking hours	**42.7**	40.7	45.1	44.2
Only for eating	**3.5**	4.4	2.8	2.3
Only in company	**0.9**	0.9	1.4	0.0
Do not use them	**4.8**	3.5	2.8	11.6

The bold numbers are for the Total columns

***p < 0.001

On this topic no significant differences were found by economic status, periphery versus center, or gender.

### Health behavior and knowledge

Two measures were used to examine the health behavior of the interviewees: The frequency of their dental visits for preventive check-ups, and the frequency of their toothbrushing. Some 40% of the interviewees reported that they went to the dentist for check-ups to identify and treat problems before these could become more severe and painful; 31.0% went at least once a year, and 8.9% went more often. Those who reportedly did not do so, were asked why not. The two main reasons given were unawareness of the importance of check-ups (63.6%) and the cost of treatment (6.7%). Differences were also found in health behavior, by economic status: 27.8% of those who found it difficult to cover their monthly expenses went for check-ups whereas 51.2% of those who had no difficulty, did so; 18.3% of the former and 3.6% of the latter said they did not go for check-ups for financial reasons (Table 6).

**Table 6:** Periodic check-ups, by ability to cover monthly expenses (%) (n = 482)

	Total^a^	No difficulty covering monthly expenses	Manage to cover monthly expenses	Find it difficult to cover monthly expenses
Frequency of periodic check-ups*				
Every 6 months	**9.9**	16.3	8.3	8.9
Once a year	**21.1**	25.6	23.7	17.7
Seldom	**8.9**	9.3	10.3	1.3
Not in the habit of seeing a dentist	**60.1**	48.8	57.7	72.2
Reasons for none, or widely-spaced check-ups*				
No time	3.6	15.0	2.4	5.5
Financial reasons	**6.7**	5.0	3.6	18.3
Waiting time/distance from clinic	**2.8**	5.0	3.0	1.7
Not considered necessary	**63.6**	50.0	67.3	55.0
Forgetfulness/laziness	**6.3**	5.0	6.5	8.3
Other	**17.0**	20.0	16.7	11.7

The bold numbers are for the Total columns

*P < 0.05

^a^Including 30 interviewees who did not answer the question on the ability to cover monthly expenses

Toothbrushing can enhance the health of teeth and gums; 69.4% of the interviewees reported that they brushed their teeth at least twice a day, 23.2%—once a day, 4%—less frequently, and 3.4%—not at all.

Another topic examined was the interviewees’ knowledge of tooth health and their attitudes towards it; 44.8% noted that eating sweets was harmful to tooth health, to a great extent, 17.2%—to a moderate extent, 9.8%—to a small extent, and 28.2% claimed that eating sweets was not harmful to tooth health or that they did not know whether it was harmful.

Various factors may impact oral health including an individual’s health behavior, its attitudes to tooth health, and their background characteristics such as education and sex. To learn of the impact of each variable, multivariate analysis was used to explain the number of natural teeth (as a measure of oral health) (Table 7). Linear regression was used for the following explanatory variables:

- Background characteristics—age group (1 = the 85+ older adults), 0 = under 85), sex (women versus men), economic status (1 = managing to cover monthly expenses, 0 = find it difficult to cover monthly expenses), population group (1 = Jews, 0 = non-Jews)
- Health behavior
  - Dental visits for preventive check-ups (versus no such visits)
  - Toothbrushing-1 = twice daily or more, 0 = less than twice a day
  - Knowledge—Sweets are harmful to teeth (1 = agreed with statement, 0 = said sweets are not harmful to teeth)

**Table 7:** Regression coefficients to explain the number of teeth of older adults

	B	SD	Beta	t	Significance
Age (85+)	− 8.314	2.007	− 0.214	− 4.142	0.000
Dental visits for preventive check-ups	4.708	1.277	0.190	3.688	0.000
Managing to cover monthly expenses	4.429	1.575	0.147	2.812	0.005
Population group (Jews)	5.239	2.292	0.119	2.286	0.023
Frequency of toothbrushing (twice daily)	2.562	1.375	0.096	1.864	0.063
Sex (women)	1.588	1.271	0.065	1.250	0.212
Knowledge (sweets are harmful to teeth)	0.125	1.262	0.005	0.099	0.921
Constant	5.354	2.788		0.615	0.539

Background characteristics and health behavior were found to impact on oral health. The variables that had a great effect on the number of teeth were:

- Negative association—age (as age rises, the number of teeth drops)
- Positive association—dental visits for preventive check-ups
- Good economic status (managing to cover monthly expenses)
- Religion (Jewish)
- Frequency of toothbrushing (the greater the frequency, the more teeth)

The model explained 15% of the variation of the dependent variable (R^2^ = 0.151; F = 8.44). The association between knowledge, sex, and number of teeth was weak. The same was true of the association between peripheral residence and number of teeth, and this variable was not entered into the regression.

## Discussion

Currently, a year into the reform of dental services, this study provides accurate data regarding the provision and distribution of dental services at the national level.

This paper presents an overall picture of aspects of oral health and health behavior among older adults aged 65+ in Israel at the start of 2020. It revealed that some 66% of this age group assessed their oral health as good or very good. This percentage is higher than that of a similar study conducted 22 years ago [6], when 54% assessed their oral health thus. The data on the number of teeth reinforce the finding on improved dental health among the 65+ age group: In 2001, 52% of the 65+ age group reported that they had lost all their teeth and the rest had an average of 10 natural teeth (a loss of 22 teeth on average). In the current study, 24% of the 65+ age group had lost all their teeth, and the rest were left with an average of 19 natural teeth. This improvement aside, the current findings show that the situation is similar to that of 15–20 years ago in developed countries such as Australia, Germany, Britain, and Sweden [9, 16], and poorer than that of the US in recent years.

The comparison of the number of teeth of Israel’s 65+ age group with that of older adults in other western countries shows that Israel’s 65–74 age group have three teeth fewer on average than their peers in the US and Australia; the 75+ age group have 4–6 teeth fewer on average [9]. Unfortunately, there are scant, up-to-date, international comparative studies on oral health, consequently the findings for Israel of 2020 had to be compared with the studies conducted in developed countries some two decades ago.

WHO has set the number of natural teeth for normal functioning at 20 to enable comfortable eating and socializing without embarrassment. This study showed that the situation in Israel is problematic as 63% of the 65+ age group reported that they had 20 teeth or fewer.

Like other studies in Israel and around the world [5, 10, 16], which found that socio-economic status and advanced age are important predictors of oral health, our study, too, found prominent differences between older adults who found it difficult to cover their monthly expenses, to assess their oral health as inferior, and have fewer teeth, on the one hand, and those who did manage to cover their monthly expenses, on the other. Of the former, 53% assessed their oral health as good or very good, and 39% reported that they had no teeth. This contrasts with the 74% and 17%, respectively, found in the two categories among those who managed to cover their monthly expenses without difficulty. The findings on the former group are apparently better than those of a study conducted among vulnerable older adults referred by social services to mobile treatment units [4]. According to the latter study, 44% assessed their oral health as good or very good, and about 50% had no teeth.

In common with the study conducted 22 years ago [6] and other studies around the world [11], which found no differences in the state of oral health between men and women, this study found a slightly better situation among women than men in the general population, although the differences were not statistically significant. The situation was also better among Jews than non-Jews, as shown by Adut [1]. As regards periphery versus center, 33% of the peripheral population had no teeth versus 25% in the center. However, the differences in the number of teeth by peripheral level, among those had teeth, were small.

One factor impacting oral health is health behavior. Periodic dental check-ups and toothbrushing were found to have a positive impact on oral health. Note that most of the older adults were not in the habit of visiting dentists for check-ups, and the overwhelming majority of them were unaware of the importance to do so. A lower percentage of the 65+ age group of an inferior economic status had preventive check-ups than the 65+ age group whose economic status was fair. A comparison with the situation of 22 years ago [6] indicates some improvement in health behavior, as reflected by both the percentage who did go for dental check-ups and the frequency of toothbrushing. The percentage of older adults brushing their teeth at least twice daily rose from 61 to 69%, and the percentage going for check-ups doubled—from 20 to 40%. These improvements might be an early positive effect of the reform. However, this should be further evaluated in the future.

These findings will hopefully serve policymakers in the promotion of the oral health of the older population in the current reforms. Nonetheless, the impact of the reform on the indicators of oral health and the use of dental services, as on the removal of barriers to service consumption, should be examined in another year or two. In addition, it is recommended that an in-depth study be conducted among Ethiopian-Israelis and Bedouin to learn about the state of oral health among them and their patterns of utilization of dental services.

### Study limitations

The data collected in our study was self-reported since no clinical examinations were conducted.

## Summary of conclusions and recommendations

Despite the improvement in oral health and oral behavior of older adults aged 65+ in the past two decades, the state of oral health in Israel remains poorer than that of other western countries. Partially, this is explained by the barriers to the consumption of dental services, including their high cost and the lack of awareness of their importance. These barriers were prominent mainly among subjects of an inferior economic status, and non-Jews. Our preliminary results are promising regarding the positive influence of the 2019 reform.

The barriers to the consumption of dental services, primarily those associated with economic status and lack of awareness, make it necessary to take steps to make services accessible with the emphasis on the 65+ age group who struggle financially, and on non-Jews. One example is the operation of a mobile dental service for residents of the periphery, the Arab population, and recipients of welfare services. In addition, publicity campaigns should be adopted concerning the importance of maintaining oral health among the 65+ age group.

Comparison of our findings with future studies will help policy makers evaluate the effectiveness of the reform and improve it in the future.

## Data Availability

N/A.

## Abbreviation

MJB: Myers-JDC-Brookdale Institute

## Footnotes

1 The first national survey on the state of health and nutrition among the 65 + age group was conducted in 2005–06 by the Israel Center for Disease Control and the Food Control Service of the Ministry of Health in cooperation with: The Israeli Society of Hypertension; the Geriatrics Department, and the Dental Health Division of Israel’s Ministry of Health; Maccabi Health Services; Clalit Health Services; AND JDC-Israel ESHEL (the association for the planning and development of services for older adults).

2 The center of the country included the districts of Jerusalem, Petah Tikva, Ramla, Rehovot and Tel Aviv. The "periphery" included Yizrael, Acre, the Golan, and Beer Sheva. The remaining districts were defined as "moderate periphery".

## References

[1] Adut R, Mann J, Sgan-Cohen HD (2004). Past and present geographic location as oral health markers among older adults. J Public Health Dent.

[2] Aarabi G, Reissmann DR, Seedorf U, Becher H, Heydecke G, Kofahl C (2018). Oral health and access to dental care—a comparison of elderly migrants and non-migrants in Germany. Ethn Health.

[3] Barmes DE, Cohen LK (1981). International collaborative study of dental manpower systems: interim report.

[4] Berg-Warman A, Zusman SP, Sasson A (2016). Dental treatment needs of vulnerable elderly in Israel—data analysis of the 'Smiling Again' project. Gerontol Geriatr.

[5] Berg-Warman A, Horev T, Zusman SP (2004). Dental health and patterns of dental service usage among the elderly in Israel. Gerontol Geriatr.

[6] Berg A, Zusman SP, Horev T. Social and economic aspects of dental care in Israel in the era of National Health Insurance. RR-359-01. Jerusalem: Myers-JDC-Brookdale Institute; 2001.

[7] Central Bureau of Statistics (CBS) 2013. The social survey 2013.

[8] Chen MS, Andersen RM, Barmes DE, Leclerq MH, Lyttle CS. Comparing oral health care systems: a second international collaborative study (No. WHO/ORH/ICSII/97.1). World Health Organization; 1997.

[9] Crocombe LA, Mejia GC, Koster CR, Slade GD (2009). Comparison of adult oral health in Australia, the USA, Germany and the UK. Aust Dent J.

[10] Friedman PK, Kaufman LB, Karpas SL (2014). Oral health disparity in older adult dental decay and tooth loss. Dent Clin North Am.

[11] Griffin SO, Griffin PM, Li C, Bailey WD, Bruson D, Jones JA (2019). Changes in older adults' health and disparities: 1999 to 2004 and 2011 to 2016. J Am Geriatr Soc.

[12] Israel Ministry of Health 2010. State of health and nutrition survey among the 65+ age group 2005–06. https://www.health.gov.il/ICDC/mabat/Pages/mabat_gold.aspx. Accessed 15 May 2020.

[13] Lee W, Kim S, Albert JM, Nelson S (2014). Community factors predicting dental utilization among older adults. J Am Assoc..

[14] Mann J, Mersel A, Gabai E (1985). Dental status and dental needs of an elderly population in Israel. Commun Dent Oral Epidemiol.

[15] Masood M, Newton T, Bakri NN, Khalid T (2017). The relationship between oral health and oral health related quality of life among elderly people in United Kingdom. J Dent.

[16] Müller F, Naharro M, Carlsson GE (2007). What are the prevalence and incidence of tooth loss in the adult and elderly population in Europe?. Clin Oral Implants.

[17] Natapov L, Kushnir D, Goldsmith R, Dichtiar R, Zusman SP (2018). Dental status, visits, and functional ability and dietary intake of elderly in Israel. Isr J Health Policy Res.

[18] National Institute of Dental Research (US). Epidemiology, & Oral Disease Prevention Program. 1988. Oral Health of United States adults: the national survey of oral health in US employed adults and seniors, 1985–1986: regional findings (No. 88). Epidemiology and Oral Disease Prevention Program, National Institute of Dental Research, US Department of Health and Human Services, Public Health Service, National Institutes of Health; 1988

[19] O’Sullivan I, Lader D, Beavan-Seymour C, Chenery V, Fuller E, Sadler K. Foundation report: adult dental health survey 2009 (technical information). Adult Dental Health Survey 2009. 2011;138. Retrieved from: http://doc.ukdataservice.ac.uk/doc/6884/mrdoc/pdf/6884foundation_report_and_technical_information.pdf.

[20] Raphael C (2017). Oral health and aging. Am J Public Health.

[21] Shnoor Y, Cohen Y (2020). The 65+ population in Israel: statistical abstract.

[22] Zusman SP, Kushnir D, Natapov L, Goldsmith R, Dichtiar R (2016). Oral health-related quality of life of the elderly in Israel—results from the national health and nutrition survey of the elderly 2005–2006. Oral Health Prev Dent.

